# Comparison of the Chemical and Technological Characteristics of Wholemeal Flours Obtained from Amaranth (*Amaranthus* sp.), Quinoa (*Chenopodium quinoa*) and Buckwheat (*Fagopyrum* sp.) Seeds

**DOI:** 10.3390/foods10030651

**Published:** 2021-03-19

**Authors:** Phara De Bock, Lori Daelemans, Lotte Selis, Katleen Raes, Pieter Vermeir, Mia Eeckhout, Filip Van Bockstaele

**Affiliations:** 1 Research Unit of Cereal and Feed Technology, Department of Food Technology, Safety and Health, Faculty of Bioscience Engineering, Ghent University, Valentin Vaerwyckweg 1, 9000 Ghent, Belgium; lori.daelemans@hotmail.com (L.D.); lotteselis@hotmail.com (L.S.); mia.eeckhout@ugent.be (M.E.); 2 Research Group VEG-i-TEC, Department of Food Technology, Safety and Health, Faculty of Bioscience Engineering, Ghent University, Gr. Karel de Goedelaan 5, 8500 Kortrijk, Belgium; katleen.raes@ugent.be; 3 Laboratory for Chemical Analysis (LCA), Department of Green Chemistry and Technology, Faculty of Bioscience Engineering, Ghent University, Valentin Vaerwyckweg 1, 9000 Ghent, Belgium; pieter.vermeir@ugent.be; 4 Food Structure and Function Research Group (FSF), Department of Food Technology, Safety and Health, Faculty of Bioscience Engineering, Ghent University, Coupure Links 653, 9000 Ghent, Belgium; filip.vanbockstaele@ugent.be

**Keywords:** pseudocereals, amaranth, quinoa, buckwheat, wholegrain, chemical composition, micronutrients, technological properties, pasting behaviour

## Abstract

A sound fundamental knowledge of the seed and flour characteristics of pseudocereals is crucial to be able to promote their industrial use. As a first step towards a more efficient and successful application, this study focuses on the seed characteristics, chemical composition and technological properties of commercially available pseudocereals (amaranth, quinoa, buckwheat). The levels of starch, fat, dietary fiber and minerals were comparable for amaranth and quinoa seeds but the protein content is higher in amaranth. Due to the high amount of starch, buckwheat seeds are characterised by the lowest amounts of fat, dietary fibre and minerals. Its protein content ranged between that of amaranth and quinoa. Buckwheat seeds were larger but easily reduced in size. The lipid fraction of the pseudocereals mostly contained unsaturated fatty acids, with the highest prevalence of linoleic and oleic acid. Palmitic acid is the most abundant unsaturated fatty acid. Moreover, high levels of P, K and Mg were found in these pseudocereals. The highest phenolic content was found in buckwheat. Amaranth WMF (wholemeal flour) had a high swelling power but low shear stability. The pasting profile strongly varied among the different quinoa WMFs. Buckwheat WMFs showed high shear stability and rate of retrogradation.

## 1. Introduction

The demand for healthy foods has increased over the last years as consumers have become more aware of the relation between diet and health [1,2]. Besides highly nutritious, cereal foods are considered to be rich in health-beneficial bioactive compounds [3,4]. The development of wholegrain products has been growing, especially as the bran and germ fraction of the grain contains high contents of nutrients and phytochemicals. Moreover, the gluten-free food market is expanding due to the higher prevalence of gluten-related disorders, such as celiac disease or gluten sensitivity. Cereals, such as wheat, maize and rice, are commonly used for the production of wholegrain and/or gluten-free products but pseudocereals are promising alternatives [5,6].

Pseudocereals are grown for the same purpose as true cereals but they are not a part of the *Gramineae* family [7]. From an ecological point of view, pseudocereals have better resistance to biotic and abiotic stresses than conventional cereal crops [8,9]. Their cultivation is possible in regions with a harsh climate and poor soil conditions, which can help to ensure food availability in these types of regions [4,10]. The most common pseudocereals are amaranth (*Amaranthus* sp.), quinoa (*Chenopodium quinoa*) and buckwheat (*Fagopyrum* sp.). The *Amaranthus* genus includes approximately 60 species but only *A. hypochondriacus*, *A. caudatus* and *A. cruentus* are cultivated as grain species. Quinoa is one of the approximately 250 species of the *Chenopodium* genus. Both sweet and bitter quinoa varieties exist, depending on the saponin content. Within the *Fagopyrum* genus, *F. tataricum* and *F. esculentum* are the most consumed and cultivated species [11,12]. Pseudocereals produce seeds with high nutritional value. The protein content of the seed is high and characterised by a well-balanced amino acid profile. The seeds are a good source of unsaturated fatty acids, dietary fibre and essential micronutrients. Furthermore, they contain a large variety of bioactive compounds [3,8,13,14]. Due to the lack of gluten, these pseudocereals are interesting ingredients for gluten-free products as well [15]. However, the presence of antinutrients may restrict the use of pseudocereals in the human diet. Some of the reported antinutrients in pseudocereals are phytic acid, saponins, tannins and protease inhibitors [9,16,17]. Several techniques of industrial processing, such as heat treatment, extrusion, roasting, or mechanical abrasion, are used to inactivate or reduce these antinutrients [16,18,19].

Pseudocereals can be used as a supplement to enrich traditional foods or for the production of new food products. Nevertheless, these pseudocereals lack dough-forming, and thus, baking properties due to the absence of gluten. The dough-forming properties of raw material are essential for all food products that demand dough preparation (bread, bakery products, pasta, etc.). Traditionally, these type of food products are made of refined wheat flour. Up to a certain amount, pseudocereal can be added to wheat-based products to improve the nutritional properties of the resulting food product. However, the addition of high amounts results in technological challenges due to the high fibre content and the dilution effect on gluten. Complete substitution of wheat comes with the challenging task of replacing the functionality of the gluten and cannot be carried out without the addition of specific ingredients or adaptations of the process conditions [6].

From a nutritional point of view, pseudocereals are promising ingredients for the production of wholegrain and/or gluten-free food products [5]. Nevertheless, the sensorial and textural properties of the final product depend on the composition of the macronutrients and the interaction of these components during processing [20]. The properties of pseudocereal wholemeal flour (WMF) to a large extent depend on the composition and properties of the starch fraction but non-starch components (i.e., protein, lipid, fibre) are also likely to have an effect. The chemical composition of pseudocereals is rather different from that of common cereals and furthermore differs among the different types of pseudocereals [5,21]. Sound fundamental knowledge of the seed and flour characteristics of pseudocereals is crucial to be able to promote their industrial use. As a first step towards a more efficient and successful application, this study focuses on the seed characteristics, chemical composition and technological properties of commercially available pseudocereals (amaranth, quinoa, buckwheat). The characteristics of the pseudocereal WMFs are discussed in relation to the seed characteristics and the chemical composition and compared among the different types of pseudocereal.

## 2. Materials and Methods

### 2.1. Material

Commercially available amaranth, quinoa and dehulled buckwheat seeds were collected from different suppliers in Belgium and the Netherlands. An overview of the pseudocereal samples is included in Table 1.

For chemical and technological analyses, grains were milled WMF by a Hammertec mill (mesh size: 0.8 mm) (Foss, Hilleroed, Denmark).

### 2.2. Characteristics of Seeds

Test weight (kg/hL) was determined by a Grain Analysis Computer (GAC 2100, Dickey-john, Auburn, AL, USA). Thousand kernel weight (TKW, g) was calculated based on the weight of 300 kernels, selected by a Contador seed counter (Pfeuffer GmbH, Kitzingen, Germany). Kernels were scanned (dpi = 600) against a blue background (HP Scanjet 2400, Hewlett-Packard Company, CA, USA). The resulting images were analysed using SmartGRAIN software (version 1.1, National Institute of Agrobiological Sciences, Tsukuba, Japan) to estimate the length (mm), width (mm) and circularity of the kernels.

### 2.3. Chemical Composition of Wholemeal Flour

#### 2.3.1. Macronutrients

Dry matter (dm) content was determined according to ICC method no. 110. Total starch content (g/100 g dm) was analysed as described by Englyst et al. [22]. The nitrogen content was determined by the use of a VarioMax C/N (Elementar Analysesystemen, Langenselbold, Germany) and converted to protein content (g/100 g dm) using a conversion factor of 6.25 [23]. Fat content (g/100 g dm) was determined by Soxhlet extraction with prior acid hydrolysis (ISO 6491). Total dietary fibre content (TDF, g/100 g dm) was quantified by the Total Dietary Fiber Assay Kit (Megazyme Ltd., Wicklow, Ireland). Ash content (g/100 g dm) was determined according to standard method ICC no. 104/1.

#### 2.3.2. Fatty Acids

Lipids were extracted from 2.5 g WMF by means of a modification of Folch et al. [24] using chloroform/methanol (2:1 *v*/*v*). The fatty acids were analysed by gas chromatography (GC) as described by Raes et al. [25]. Briefly, nonadecanoic acid (C19:0) was added as an internal standard (2 mg/mL). After methylation of the fatty acids with NaOH/MeOH followed by HCl/MeOH, the fatty acid methyl esters (FAME) were analysed on an Agilent 6890 using a DB-23 column for FAME (60 m × 0.25 mm × 0.15 µm) (Agilent, Santa Clara, CA, USA). The GC conditions were: injector: 250 °C; detector: 280 °C; He as carrier gas; temperature program: 50 °C for 1 min, followed by an increase of 25 °C/min to 175 °C, then 4 °C/min to 230 °C.

#### 2.3.3. Minerals

The mineral composition was determined via inductive coupled plasma-optical emission spectrometry (ICP-OES, IRIS Intrepid II XSP, Thermo Scientific, Waltham, MA, USA). Prior to analysis, 1.0 g of WMF was ashed at 500 °C for 4 h in a muffle furnace. The ash was dissolved in HCl during a 2 h reflux. The remaining residue was filtered over a Whatman filter no. 5 before analysis.

#### 2.3.4. Phenolic Compounds

Soluble and bound phenolic compounds (PC) were extracted according to Shumoy et al. [26]. Briefly, 5 g of WMF were weighed into a 50 mL test tube and homogenised with methanol (15 mL, 100%) at 10,000 rpm for 45 s using an Ultra Turrax (T18 digital, IKA-Werke GmbH & Co. KG, Staufen, Germany). The tubes were placed on ice for 15 min and subsequently centrifuged at 4000× *g* for 15 min at 4 °C. The residue was re-extracted using 10 mL 80% methanol, following the same procedure. Both supernatants were filtered over a filter paper no. 413 (VWR, Leuven, Belgium) and collected in a 25 mL volumetric flash. The volume was corrected to 25 mL using 80% methanol (=extract of soluble PC).

After removal of the supernatant, the pellet was dried overnight at room temperature. 0.1 g of the dried pellet were hydrolysed using 2 mL of 2 M NaOH and sonicated (UP 400s, dr. Hielscher, GmbH, Hamm, Germany) at maximum amplitude (100%) for 30 min at 60 °C. The solution was neutralised by the addition of HCl. Then, 4 mL of 100% methanol containing 0.1% (*v*/*v*) formic acid was added as an extraction solvent. The solution was vortexed for 2 min and centrifuged at 2000× *g* for 10 min at 4 °C. The pellet was re-extracted using 4 mL of 100% methanol containing 0.1% (*v*/*v*) formic acid, following the same procedure. Both supernatants were filtered (filter paper no. 413, VWR, Leuven, Belgium) and collected in a 20 mL volumetric flash. Volume was adjusted to 20 mL using 80% methanol (=extract of bound PC).

The total PC content was determined by the method described by Shumoy et al. [26]. Briefly, 1 mL of diluted soluble or bound extract was mixed with 0.5 mL of 10 times diluted Folin–Ciocalteu’s reagent, vortex mixed and allowed to stand for 6 min. The reaction was neutralised by the addition of 1.5 mL 20% sodium carbonate solution and 1 mL of double-distilled water. After 2 h in the dark, the absorbance of the solution was measured at 760 nm using a spectrophotometer (4001/4, Thermo Spectronic, Waltham, MA, USA). Gallic acid was used as a standard and the results were presented as mg gallic acid equivalents (GAE)/g dm.

High-performance liquid chromatography (HPLC) profiling of the PC was done with an Agilent 1260 Infinity II (Agilent, Santa Clara, CA, USA) equipped with a NUCLEODUR 100-5 C18 ec column (particle size: 5 µm, internal diameter: 4.6 mm, length: 150 mm) (Macherey-Nagel, Düren, Germany). HPLC-grade water and methanol were used as mobile phase A and B, respectively, with a flow rate of 0.5 mL/min. The gradient conditions were set as: 0–5 min, 25% B; 5–10 min: 25–30% B; 10–16 min: 30–45% B; 16–18 min: 45% B; 18–25 min: 45–80% B; 25–30 min: 80% B and 30–35 min: 80–25% B. The injection volume was 20 µL. The detection wavelengths of the diode array detector (DAD) were set at 254, 275, 305, and 320 nm [27]. Identification of the PhC was done by comparing retention times and spectra from the DAD detector with those of pure standards. The wavelengths used for the quantification were: 254 nm for gallic acid, dihydroxybenzoic acid, vanillic acid, o-coumaric acid, rutin, quercetin and kaempferol; 275 nm for catechin, p-coumaric acid and sinapic acid; and 305 nm for caffeic and ferulic acid.

### 2.4. Technological Properties of Wholemeal Flour

The α-Amylase activity was quantified by the α-Amylase Assay Kit (K-CERA, Megazyme Ltd., Wicklow, Ireland). One Ceralpha Unit (CU) is defined as the amount of enzyme, in the presence of excess thermostable α-glucosidase, required to release one micromole of p-nitrophenol from BPNPG7 in one minute under the defined assay conditions. Starch damage (% dm) was quantified by the Starch Damage Assay Kit (K-SDAM, Megazyme Ltd., Wicklow, Ireland).

Water absorption and swelling power were determined as followed. An aqueous suspension of 0.075 g WMF in 1.5 mL distilled water was shaken for 30 min at 1000 rpm in a ThermoMixer C (Eppendorf, Hamburg, Germany). The shaking temperature was 21 °C to determine the water absorption. The swelling power was determined at a shaking temperature of 55, 65, 75, 85 and 95 °C. Subsequently, the suspension was centrifuged for 20 min at 8000× *g* and 21 °C. The supernatant was decanted and the sediment was weighed. Water absorption (g/g) was expressed as the amount of water absorbed by the WMF. Swelling power (g/g) was calculated as the weight of sediment per gram of WMF used.

Pasting properties of the WMF were determined using a Rheometer MCR 102 (Anton Paar GmbH, Graz, Austria). Measurement was performed using 2.8 g WMF (based on 14% moisture) dispersed in 20 mL of distilled water. During the pre-shear phase, the suspension was heated to 50 °C while stirred at 960 rpm. The rotation speed was 160 rpm for the remainder of the test. The temperature was initially maintained at 50 °C for 1 min and then raised to 95 °C at a constant rate of 5 °C per min, held at 95 °C for 5 min, cooled to 50 °C at the same rate and finally held at 50 °C for 2 min [28].

### 2.5. Statistical Analysis

Data analysis was performed with SPSS Statistics 25 (SPSS Inc., Chicago, IL, USA) and R version 4.0.2 (R Core Team, Vienna, Austria). Results are represented as minimum–maximum and/or mean ± standard deviation. Experimental data were analysed by the use of one-way analysis of variance (ANOVA, *p* < 0.05) with subsequent Tukey’s test as post hoc analysis (*p* < 0.05). If homogeneity of variances was violated, a Welch ANOVA (*p* < 0.05) was used instead. In that case, a Games-Howell test (*p* < 0.05) was carried out as a post hoc analysis. Pearson tests were performed to establish correlations between different variables.

## 3. Results and Discussion

### 3.1. Characteristics of Seeds

Table 2 presents the seed characteristics of amaranth, quinoa and buckwheat. Among the pseudocereals studied, amaranth had the smallest seeds, which is indicated by the low TKW (0.69 ± 0.05 g). TKW correlated negatively with the protein (*r* = −0.806, *p* = 0.016) and fibre (*r* = −0.894, *p* = 0.003) content of the amaranth seed. Proteins are mainly located in the seed embryo which suggests that the embryo proportion is higher in amaranth seeds with a low TKW [4,29]. Nevertheless, the ash (*r* = 0.898, *p* = 0.002) and fat (*r* = 0.908, *p* = 0.002) content of the seed increased with the TKW. As minerals and fat are also stored in the embryo [29], it is assumed that not the proportion but the composition of the embryo is to a large extent determinative for the TKW of amaranth.

The seed size of quinoa is larger compared to amaranth, hence the higher TKW. Values ranged between 2.19 and 4.05 g, which is in accordance with the findings of Wu et al. (1.8–4.1 g) [30]. The TKW of quinoa increased with a decrease in the ash content (*r* = −0.799, *p* = 0.031). This TKW increase was particularly associated with a lower Ca (*r* = −0.776, *p* = 0.039), K (*r* = −0.949, *p* = 0.001) and Na (*r* = −0.901, *p* = 0.006) content. Minerals such as Ca and K are mainly located in the pericarp and seed coat of the quinoa seed. Smaller seeds, with lower TKW, have relatively more pericarp and seed coat, which results in a higher Ca and K content [31,32]. Less is known about the location of Na within the quinoa seed but these results suggest that Na might be located in the outer layers as well.

Two buckwheat samples, namely BU2 and BU7, were smaller compared to the other samples and had a TKW at the lower bound of the range (18.24 and 17.59 g, respectively). Less variation in size and weight was observed for the other eight samples. Their TKW ranged between 19.26 and 25.64 g, which is in accordance with other studies (15.70–32.57 g) [33,34]. Buckwheat seeds are larger and heavier than amaranth or quinoa seeds. The high starch content of buckwheat (Section 3.2.1) is an indication that its embryo proportion is smaller than that of the other two pseudocereals [14]. The amount of lipids, dietary fibre and minerals are also lower due to a large amount of starch.

Despite their low TKW, amaranth seeds were characterised by the highest test weight (85.2 ± 0.7 kg/hL). Even higher values (90.8 to 91.3 kg/hL) have been reported by Temesgen et al. [35]. Test weight is related to the true density and packing efficiency of the seeds [36]. Amaranth appears to have a higher true density compared to quinoa or buckwheat [37,38,39], while the small size of the amaranth seeds might attribute to a better packing efficiency.

The two red quinoa samples in the set (i.e., QU1 and QU5) stood out due to their test weight. The test weight of sample QU5 (66.8 kg/hL) was considerably lower compared to other samples (75.4–79.2 kg/hL). The low protein and fat content were typical for this sample as well (Section 3.2.1). These components are mainly stored in the embryo of the seed [6], which suggests that the embryo portion of the QU5 seeds is small, especially if compared to the other quinoa samples. The study by Gargiulo et al. [40] stated that the embryo of the quinoa seed has a higher density compared to the perisperm. The smaller embryo to perisperm ratio explains why sample QU5 had a lower test weight. Furthermore, sample QU1 transcended the other quinoa samples by its high test weight (86.1 kg/hL) and protein content. The protein (*r* = 0.971, *p* < 0.001) and fat (*r* = 0.846, *p* = 0.016) content of the quinoa seeds correlated positively with the test weight, which confirms that the portion of the embryo is determinative for the test weight. The study by Wu et al. [30] confirms the large variation in the test weight of quinoa seeds (63–81 kg/hL).

Buckwheat had an average test weight of 78.6 ± 1.1 kg/hL, which is comparable to quinoa. Studies by Stempińska et al. [34] and Parde et al. [41] reported lower values for different buckwheat varieties (58.4–70.5 kg/hL). Zhu et al. [42] studied the test weight with different methods and at different levels of seed moisture. These results (72.2–80.1 kg/hL) [42] were more within the range of the present study.

Amaranth seeds (0.88 ± 0.01) were more circular compared to quinoa seeds (0.85 ± 0.02), while Medina et al. [43] concluded the opposite. Other studies also reported lower values for the circularity of amaranth (0.81–0.83) [37] and quinoa (0.77–0.80) [38]. Buckwheat had the lowest circularity (0.76 ± 0.02), which is predictable as these kernels are triangular in shape. Amaranth and quinoa kernels both have a lenticular shape which explains the higher circularity.

### 3.2. Chemical Composition of Wholemeal Flour

#### 3.2.1. Macronutrients

The proximate compositions of amaranth, quinoa and buckwheat WMF are presented in Table 3. The pseudocereals had a starch content of 61.0 ± 2.9 g/100 g dm for amaranth, 65.1 ± 6.3 g/100 g dm for quinoa and 70.5 ± 3.1 g/100 g dm for buckwheat. Buckwheat shows a higher starch level due to a greater proportion of the endosperm material [14]. Buckwheat sample BU4 was characterised by the highest starch content, namely 78.3 g/100 g dm. Torbica et al. [44] reported a similar starch content (80.4 g/100 g dm) for husked buckwheat flour. Quinoa samples showed great variation in starch content, as concluded by Li et al. (58.2–67.6 g/100 g dm) [21] as well.

Amaranth was characterised by the highest protein content (16.0 ± 0.5 g/100 g dm). Previous studies have reported 14.8 to 17.8 g protein/100 g dm in amaranth [4,45]. Sample BU4, being high in starch, had the lowest protein content (13.9 g/100 g dm) among the buckwheat samples. Unal et al. [46] also found a protein content of 13.8 g/100 g dm. For the other buckwheat samples, the amount of proteins varied between 14.4 and 16.4 g/100 g dm, which is within the range of results from other studies (14.0–17.9 g/100 g dm) [14,45,46,47]. In comparison to amaranth and buckwheat, most quinoa samples had a lower protein content (11.9–14.0 g/100 g dm, QU5: 9.5 g/100 g dm). Nevertheless, sample QU1 had the highest protein content, namely 16.7 g/100 dm. Repo-Carrasco-Valencia et al. [48] also observed a wide variation in the protein content of quinoa (12.6–16.1 g/100 g dm).

Both amaranth (6.81 ± 0.29 g/100 g dm) and quinoa (6.36 ± 1.65 g/100 g dm) contained high amounts of fat. This is attributed to the high proportional size of the embryo within these seeds [14]. Values are in accordance with previous studies on amaranth (5.7–6.9 g/100 g dm) [4] and quinoa (3.6–8.1 g/100 g dm) [21,48,49]. The fat content of QU5 (2.74 g/100 g dm) was considerably lower compared to the other quinoa samples. Buckwheat had the lowest fat content (3.68 ± 0.13 g/100 g dm) of all pseudocereals although lower values (1.0–2.5 g/100 g dm) have been reported in previous studies [50,51].

High amounts of TDF were found in red quinoa WMF (QU1: 13.64 g/100 g dm, QU5: 15.31 g/100 g dm) but other quinoa samples were also a good source of dietary fibre (7.15–10.67 g/100 g dm). Li et al. [21] found a similar variation in the TDF content of seven quinoa samples (8.8–17.4 g/100 g dm). Sample AM5 (6.53 g/100 g dm) contained a lower amount of dietary fibre compared to the other amaranth samples (9.27–11.16 g/100 g dm). Previous works reported TDF contents between 8.6 and 15.8 g/100 g dm for amaranth [5,52,53,54,55]. The TDF content negatively correlated with the fat (*r* = −0.834, *p* = 0.010) and ash (*r* = −0.765, *p* = 0.027) content of the amaranth WMFs. The lowest amounts of dietary fibre were found in buckwheat WMF (4.93 ± 0.68 g/100 g dm). Nevertheless, Lu et al. [56] concluded that the TDF of ten buckwheat varieties varied between 3.6 and 10.6 g/100 g dm.

Amaranth (2.53 ± 0.20 g/100 g dm) and quinoa (2.62 ± 0.59 g/100 g dm) had a comparable ash content, as concluded by Nascimento et al. [57] as well. The ash content of amaranth was within the same range as the content in the study performed by Bojórquez-Velázquez et al. (2.8–3.5 g/100 g dm) [4]. The ash content in amaranth WMF was related to the fat (*r* = 0.892, *p* = 0.003) and protein (*r* = −0.827, *p* = 0.011) content. The ash content of quinoa varied within a wide range of 1.92 to 3.46 g/100 g dm, which is comparable to previous studies (1.9–3.5 g/100 g dm) [21,49,58,59]. Buckwheat had a lower ash content (2.12 ± 0.13 g/100 g dm) but values were in accordance with Lu et al. (1.7–2.7 g/100 g dm) [56]. A negative correlation was found between the protein and ash content (*r* = −0.683, *p* = 0.029) for the buckwheat samples.

#### 3.2.2. Fatty Acids

The fatty acid composition of amaranth, quinoa and buckwheat WMF is presented in Table 4 as percentages of the total FAME. The lipid fraction of the pseudocereals mostly contained unsaturated fatty acids. The most abundant fatty acid is linoleic acid (C18:2 n-6), followed by oleic acid (C18:1 c9). The highest levels of linoleic acid are found in amaranth (46.3 ± 1.0%) and quinoa (49.7 ± 3.5%). Previous studies reported 39.4 to 49.1% for amaranth [60] and 46.7 to 49.6% for quinoa [61,62]. The levels of linoleic acid in buckwheat WMF were in accordance with Dziadek et al. (37.6–40.9%) [50].

The level of oleic acid was comparable for amaranth (23.1 ± 1.0%) and quinoa (23.8 ± 3.2%) but lower as compared to buckwheat (33.9 ± 0.8%). Even higher levels of oleic acid have been reported for buckwheat (41.0–42.2%) [50].

The level of other unsaturated fatty acids was low in amaranth (≤1.1%), while quinoa contained considerable amounts of α-linolenic acid (C18:3 n-3, 6.76 ± 1.58%). The levels of eicosenoic acid (C20:1, 1.57 ± 0.11%) and erucic acid (C22:1, 1.51 ± 0.32%) were also higher in quinoa. The highest content of eicosenoic acid was found in buckwheat (2.85 ± 0.08%).

Palmitic acid (C16:0) is the main saturated fatty acid in the lipid fraction of pseudocereals of which amaranth contains the highest levels (18.9 ± 0.2%). Previous studies reported up to 32.6% of palmitic acid in amaranth [60,63]. The lowest level of palmitic acid was found in quinoa (9.8 ± 0.5%), while buckwheat contained 14.3 ± 0.6%. Higher levels (14.9–16.6%) have been reported for buckwheat [50].

Other saturated fatty acids are less abundant compared to palmitic acid. The level of myristic acid (C14:0) is below 1% in all pseudocereals, as is the content of stearic acid (C18:0) in quinoa WMF. Higher levels of stearic acid were found in buckwheat (1.86 ± 0.08%) and amaranth (3.53 ± 0.17%). Previous studies reported higher values for quinoa (0.84–0.94% C18:0) [61] and buckwheat (2.49–4.09% C18:0) [50].

#### 3.2.3. Minerals

Amaranth, quinoa and buckwheat WMF contained high levels of P, K and Mg (Table 5). The most abundant mineral in amaranth WMF was P. Its content ranged between 4433 and 5889 mg/kg dm, which is similar to quinoa (4287–5738 mg/kg dm) and buckwheat (4546–5515 mg/kg dm). Samples QU5 and BU10 were characterised by a lower P content of 2513 and 1687 mg/kg dm, respectively. These values are lower compared to previous studies on quinoa (3780–5951 mg/kg dm) [57,64] or buckwheat (3189–4140 mg/kg dm) [14,46,64]. It is known that the majority of P occurs as phytic acid within the pseudocereal seed. Due to its high chelating activity, phytic acid inhibits the absorption of minerals (i.e., Ca, Fe, Mg, Mn, Zn) during digestion and is, therefore, considered an anti-nutritional factor. Phytic acid can be enzymatically degraded by phytase, thereby improving mineral bioavailability [14,65]. According to Egli et al. [66], the endogenous phytase activity is relatively high in pseudocereals (0.6–2.9 phytase units/g dm). Nevertheless, the optimal conditions for the degradation of phytic acid are rarely reached during conventional processing conditions [14].

The highest levels of K were found in quinoa (8906 ± 2369 mg/kg dm), followed by buckwheat (5174 ± 1079 mg/kg dm) and amaranth (4740 ± 260 mg/kg dm). Moreover, K was the most abundant mineral in quinoa and buckwheat WMF. Like the P content, the K content of BU10 (2130 mg/kg dm) was also lower compared to other buckwheat samples. Nevertheless, similar levels of K were reported by Khan et al. (2688–2935 mg/kg dm) [51].

Amaranth (2755 ± 100 mg/kg dm) and buckwheat (2439 ± 510 mg/kg dm) contained high levels of Mg. Among the buckwheat samples, BU10 had the lowest Mg content (1016 mg/kg dm) but this is still higher compared to the findings of Bhinder et al. (737–964 mg/kg dm) [20]. The Mg content was lower in quinoa WMF (2054 ± 529 mg/kg dm) with exception of samples QU3 (2824 mg/kg dm) and QU4 (2569 mg/kg dm). Previous studies reported 1744 to 2885 mg Mg/kg dm in quinoa [21,58,64,67].

Important amounts of Ca (1930 ± 79 mg/kg dm) were found in amaranth WMF, as also confirmed by previous studies (1844–2703 mg/kg dm) [57,64,68,69]. QU3 and QU4 also had a high Ca content of 1041 and 930 mg/kg dm, respectively. Other quinoa samples contained less Ca (347–497 mg/kg dm), which is in accordance with other studies (402–882 mg/kg dm) [21,58]. The lowest levels of Ca were found in buckwheat WMF (220 ± 28 mg/kg dm).

The Na content of the different WMFs ranged between 65.0 ± 16.9 (buckwheat) and 127.1 ± 51.2 mg/kg dm (quinoa). However, pseudocereals are generally not considered a good source of Na [14]. Some studies even reported that the Na content is below the limit of quantification (LoQ: 1 mg/kg [57] and 25 mg/kg [64]). Nevertheless, the present findings for buckwheat seem to show a low Na content compared to other findings (742–1134 mg Na/kg dm) [46,51]. Genetic variability and differences in growing conditions might be attributed to the differences among studies. Processing techniques, such as dehulling, soaking, washing or polishing, might also have an impact on the mineral composition [21,57].

Pseudocereal WMF also contained important amounts of other minerals, such as Cu, Fe, Mn and Zn. The highest levels of Fe (82.6 ± 6.2 mg/kg dm), Mn (30.0 ± 3.6 mg/kg dm) and Zn (35.5 ± 5.5 mg/kg dm) were found in amaranth WMF. Especially sample AM5 showed a high Mn and Zn content of 38.1 and 47.7 mg/kg dm, respectively. The Zn content of quinoa (36.0 ± 6.2 mg/kg dm) was comparable to that of amaranth. Sample QU5 had a slightly lower Zn content (25.0 mg/kg dm) but was still within the range of previous studies (24.6–50.3 mg/kg dm) [7,21,58,69]. Furthermore, quinoa contained the highest amount of Cu and samples QU3 (129.9 mg/kg dm) and QU4 (113.1 mg/kg dm) showed high levels of Fe. Buckwheat had the lowest levels of Cu, Fe, Mn and Zn, as the overall ash content was also lower compared to those of amaranth and quinoa (Section 3.2.1). Especially sample BU10 contained a low amount of Fe (18.5 mg/kg dm) and Mn (6.2 mg/kg dm).

#### 3.2.4. Phenolic Compounds

Table 6 presents the phenolic content of the different pseudocereal WMFs. The highest content of soluble PC was found in buckwheat WMF and ranged from 1.06 to 1.28 mg GAE/g dm. The soluble phenolic content of BU9 (0.48 mg GAE/g dm) was lower compared to the other buckwheat samples. Amaranth and quinoa WMFs had a soluble phenolic content of 0.06 ± 0.01 and 0.33 ± 0.02 mg GAE/g dm, respectively. Li et al. [21] reported that the soluble phenolic content of quinoa ranged between 0.18 and 0.48 mg GAE/g with the highest content of soluble PC found in coloured quinoa samples. Values are in accordance with the present study but the soluble phenolic content of the red quinoa samples was not considerably higher compared to the other samples.

The content of bound PC was high in buckwheat WMF as well and varied between 3.06 and 3.68 mg GAE/g dm. The content of bound PC ranged between 2.26 and 2.96 mg GAE/g dm for most quinoa samples, which is comparable to amaranth WMF (2.53 ± 0.34 mg GAE/g dm). The red quinoa samples, QU1 and QU5, had a higher content of 3.97 and 3.24 mg GAE/g dm, respectively. According to Abderrahim et al. [70], the bound phenolic content of red quinoa samples varied between 1.28 to 4.52 mg GAE/g. Authors attributed this higher bound phenolic content to the high pigment content of the coloured quinoa samples [70].

The high soluble and bound phenolic content of buckwheat WMF resulted in the highest total phenolic content (4.45 ± 0.19 mg GAE/g dm). Other studies also concluded that buckwheat had a higher phenolic content compared to amaranth and quinoa [14,71]. Guo et al. [72] reported that the soluble phenolic content (8.20–16.3 mg GAE/g dm) was considerably higher compared to the bound phenolic content (0.12–0.67 mg GAE/g dm) in Tartary buckwheat. Within the present study, the bound PC contributed 72.2 to 88.5% to the total phenolic content, which is higher compared to the share of the soluble PC (13.0–38.4%). The bound phenolic content in amaranth and quinoa WMF was higher than the soluble phenolic content as well. Li et al. [21] also concluded that the bound PC were the majority of the total PC in quinoa. However, Repo-Carrasco-Valencia et al. [48] reported that the percentage share of soluble PC in quinoa ranged between 7 and 61%. Within the present study, these percentages ranged between 8.1 and 15.5%.

The determination of the soluble phenolic profile (Table 7) showed the presence of phenolic acids, such as gallic, dihydroxybenzoic, vanillic and caffeic acid, in amaranth WMF. Dihydroxybenzoic acid (0.151 ± 0.013 mg/g dm) was the most prominent phenolic acid and was present in all amaranth samples. The levels of gallic (0.014 ± 0.003 mg/g dm), vanillic (0.005 ± 0.000 mg/g dm) and caffeic acid (0.004 ± 0.001 mg/g dm) were lower in contrast to dihydroxybenzoic acid. These phenolic acids were not detected in all samples. Paśko et al. [73] found gallic, vanillic and p-coumaric acid in *Amaranthus cruentus* but did not detect caffeic acid.

The phenolic acids found in quinoa WMF were similar to those found in amaranth WMF. Caffeic and vanillic acid, however, were not detected in all quinoa samples. The level of dihydroxybenzoic acid (0.056 ± 0.034 mg/g dm) was lower compared to amaranth WMF. Repo-Carrasco-Valencia et al. [48] also reported the presence of soluble ferulic and p-coumaric acid in quinoa but these phenolic acids were not detected within the present study. Furthermore, the concentration of soluble rutin varied between 0.033 and 0.110 mg/g dm. This flavonoid was not found in the other pseudocereals, although previous studies reported its presence in amaranth [74] and buckwheat [72,75].

Buckwheat WMF contained the following phenolic acids: gallic, dihydroxybenzoic, vanillic and o-coumaric acid. Guo et al. [72] also reported the presence of ferulic and p-coumaric acid in common buckwheat, and, furthermore, detected the flavonoids rutin, quercetin and catechin.

Less PC were identified in the bound phenolic fraction of the pseudocereals but the detected PC were present in higher concentrations (Table 8). The most prominent bound compound was vanillic acid. The highest levels were found in amaranth WMF (0.447 ± 0.102 mg/g dm). Other phenolic acids or flavonoids were not detected in the bound PC fraction of amaranth. Bound vanillic (0.342 ± 0.158 mg/g dm) and caffeic (0.136 ± 0.019 mg/g dm) acid were found in quinoa WMF but these phenolic acids were not present in all samples. Only buckwheat WMF contained o-coumaric acid. The content ranged between 0.191 and 0.223 mg/g dm. This phenolic acid, however, was not found in sample BU9. The levels of vanillic (0.228 ± 0.065a mg/g dm) and caffeic (0.113 ± 0.007 mg/g dm) acid in buckwheat WMF were similar to those in quinoa WMF.

### 3.3. Technological Properties of Wholemeal Flour

#### 3.3.1. α-Amylase, Starch Damage and Water Absorption

The α-amylase activity and level of starch damage were characterised, as well as the water absorption of the WMFs (Table 9). The highest α-amylase activity was found in amaranth WMF (0.15 ± 0.06 CU/g dm), which may have an impact on the technological properties of this flour. Kaur et al. [8] studied the pasting behaviour of several *A. hypochondriacus* and *A. caudatus* lines and calculated the α-amylase activity from the difference in peak viscosity measured in the presence and absence of AgNO_3_. Inactivation of α-amylase increased the peak viscosity for *A. hypochondriacus*, while no difference was observed for *A. caudatus*. The *A. hypochondriacus* lines showed a wider and higher α-amylase activity which might explain the lower viscosity of these lines [8]. No significant correlations with the pasting properties were observed within the present study. It is possible that the α-amylase activity was too low to have an impact on the pasting behaviour, similar to what was observed for the *A. caudatus* lines in the study by Kaur et al. [8]. However, the difference in measuring technique does not allow one to compare the activities of both studies.

The α-amylase activity ranged between 0.05 and 0.11 CU/g dm for most quinoa samples. Other studies reported an activity of 0.04 CU/g [76] to 0.09 CU/g [77]. QU4 and QU5 showed higher activity of 0.32 and 1.07 CU/g dm, respectively. Aluwi et al. [78] suggested that the persisting trend of low pasting temperature, peak and final viscosity of quinoa variety Temuco was attributed to a higher α-amylase activity. Indeed, QU4 and QU5 showed a lower peak and final viscosity but this was also the case for sample QU1 with an activity of only 0.11 CU/g dm. Furthermore, no significant correlations between the pasting parameters and α-amylase activity were observed. A re-measurement of the pasting profile, in the presence of AgNO_3_ to inactivate α-amylase [8], could ensure more clarity about the impact of the high α-amylase activity in samples QU4 and QU5. However, Elgeti et al. [76] found a high α-glucosidase activity (approximately 9 units/g) in quinoa WMF and suggested that this enzyme had a greater impact on starch hydrolysis.

The lowest α-amylase activity was measured in buckwheat WMF and ranged between 0.03 and 0.10 CU/g dm. Phiarais et al. [79] reported an activity of 0.1 CU/g.

The dry-milling technique used in the WMF production caused physically damaged some of the starch granules [5]. The level of starch damage varied between 3.10 and 3.95% dm for amaranth (Table 9). This is considerably lower compared to the findings of Srichuwong et al. (11.7% dm) [5]. Nevertheless, the differences in starch damage may be caused by the different milling techniques applied [14]. The level of starch damage related positively to the TKW (*r* = 0.794, *p* = 0.019) of the amaranth seeds. Thus, the milling of larger seeds resulted in more starch damage. The amount of starch damage was higher in quinoa WMF, which contrasts with the findings of Srichuwong et al. [5]. Values ranged between 3.51 and 4.44% dm. Other studies reported higher levels of 5.4 and 10.6% dm [5,14]. The level of starch damage in buckwheat WMF was considerably lower compared to quinoa WMF, which is confirmed by the study of Hager et al. [14]. Torbica et al. [44] attributed the low level of starch damage to the less compact structure of the buckwheat seed. In other words, buckwheat seeds are more easily reduced in size during the milling process. The buckwheat samples contained between 0.94 and 1.43% dm damaged starch, which is lower compared to the findings of Hager et al. (3.0% dm) [14].

Water absorption is the ability of the WMF to physically hold water while exposed to a centrifugal force [80]. Collar et al. [81] concluded that amaranth and quinoa flour had a similar water absorption, while the water absorption of buckwheat flour was lower. Within the present study, amaranth WMF was characterised by the highest water absorption (Table 9). The high capacity to absorb water might be attributed to the high protein content. It is known that the ability of flour to associate with water, at room temperature, mainly depends on proteins [78,82]. The absorption ranged between 1.86 and 2.15 g/g, which is in accordance with previous studies (1.60–2.15 g/g) [82,83]. Amaranth samples with a high water absorption capacity were usually characterised by a lower peak viscosity (*r* = −0.812, *p* = 0.014) and holding strength (*r* = −0.788, *p* = 0.020).

The water absorption of quinoa WMF varied from 1.52 to 1.74 g/g, which is higher compared to the findings of Aluwi et al. (0.89–1.22 g/g) [78]. Sample QU5 was characterised by high water absorption of 2.05 g/g. Water absorption correlated positively with the α-amylase activity (*r* = 0.929, *p* = 0.002), which indicates that the high absorption capacity of QU5 is attributed to its high α-amylase activity. This is unexpected as the enzymatic hydrolysis of starch is known to reduce water absorption [84].

The water absorption capacity of buckwheat (1.67 ± 0.06 g/g) was comparable to quinoa. Higher values were reported (2.25–2.73 g/g) [20]. The water absorption of buckwheat WMF related positively with the starch (*r* = 0.686, *p* = 0.029) and TDF (*r* = 0.718, *p* = 0.019) content of the flour, while a lower protein content (*r* = −0.697, *p* = 0.025) was associated with a higher absorption capacity. This indicates that starch and dietary fibre are the main hydrophilic constituents, attributing to the water absorption capacity of buckwheat WMF. Water absorption correlated positively with the swelling power at 95 °C (*r* = 0.835, *p* = 0.003) and important pasting parameters, such as peak viscosity (*r* = 0.882, *p* = 0.001), holding strength (*r* = 0.916, *p* < 0.001) and final viscosity (*r* = 0.965, *p* < 0.001).

#### 3.3.2. Swelling Power

**Figure 1:**
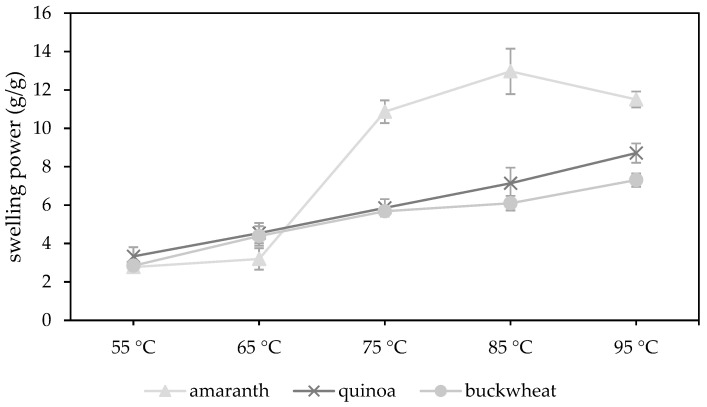
Swelling power (g/g) of amaranth (*n* = 8), quinoa (*n* = 7) and buckwheat (*n* = 10) wholemeal flour measured at different temperatures (55, 65, 75, 85 and 95 °C).

The swelling behaviour of amaranth, quinoa and buckwheat WMF were characterised by measuring the swelling power at 55, 65, 75, 85 and 95 °C (Figure 1). The data table is presented in the Supplementary Materials (Table S1). Each pseudocereal had a different swelling behaviour, which indicates differences in the molecular organization of the starch granules [85]. The swelling power of a starch granule is attributed to its strength and micellar structure and is in fact a property of amylopectin [86,87].

The swelling power of amaranth WMFs showed a strong increase from 3.18 ± 0.56 to 10.87 ± 0.59 g/g when temperature increased from 65 to 75 °C. Siwatch et al. [88] also reported a strong increase in the swelling power from 60 to 70 °C for amaranth starch. Furthermore, the swelling power reached a maximum at 85 °C (12.97 ± 1.19 g/g) and eventually dropped to 11.50 ± 0.42 g/g at 95 °C. The temperature increase causes damage to the starch granules, which enables the granules to absorb more water [86]. At peak temperature, the rate of starch swelling is equal to the rate of starch disintegration [69]. As such, the swelling power of amaranth WMF increased as long as the temperature was reaching its peak temperature (84.25 ± 0.91 °C, Section 3.3.3) but decreased at 95 °C. This decrease is attributed to the increased solubility and leaching out of more solids [86]. Quinoa (95.07 ± 0.11 °C) and buckwheat (95.08 ± 0.03 °C) WMFs had a higher peak temperature causing a continued increase of the swelling power at 95 °C.

The swelling power of quinoa WMF showed a steady increase from 3.33 ± 0.47 g/g at 55 °C to 8.71 ± 0.50 g/g at 95 °C. Similar results were reported by Li et al. [21]. The swelling power of buckwheat followed a similar trend from 55 to 75 °C but showed a less strong increase from 75 to 95 °C. The high amylose content of buckwheat starch (21.1–46.6%) might attribute to the lower swelling power at temperatures above 75 °C [10]. Amylose reinforces the internal structure within the starch granule, which renders the granule more resistant to swelling. The presence of amylose-lipid complexes can also inhibit the swelling and solubilisation of the starch granule as it prevents the amylose from leaching out [87]. In amaranth starch, the amylose content is much lower (0.1–11.1%) and the inhibitory effects of amylose-lipid complexes are absent or negligible [5,6]. This explains why amaranth WMFs showed a much higher swelling power at temperatures above 65 °C compared to quinoa or buckwheat WMF.

#### 3.3.3. Pasting Properties

**Figure 2:**
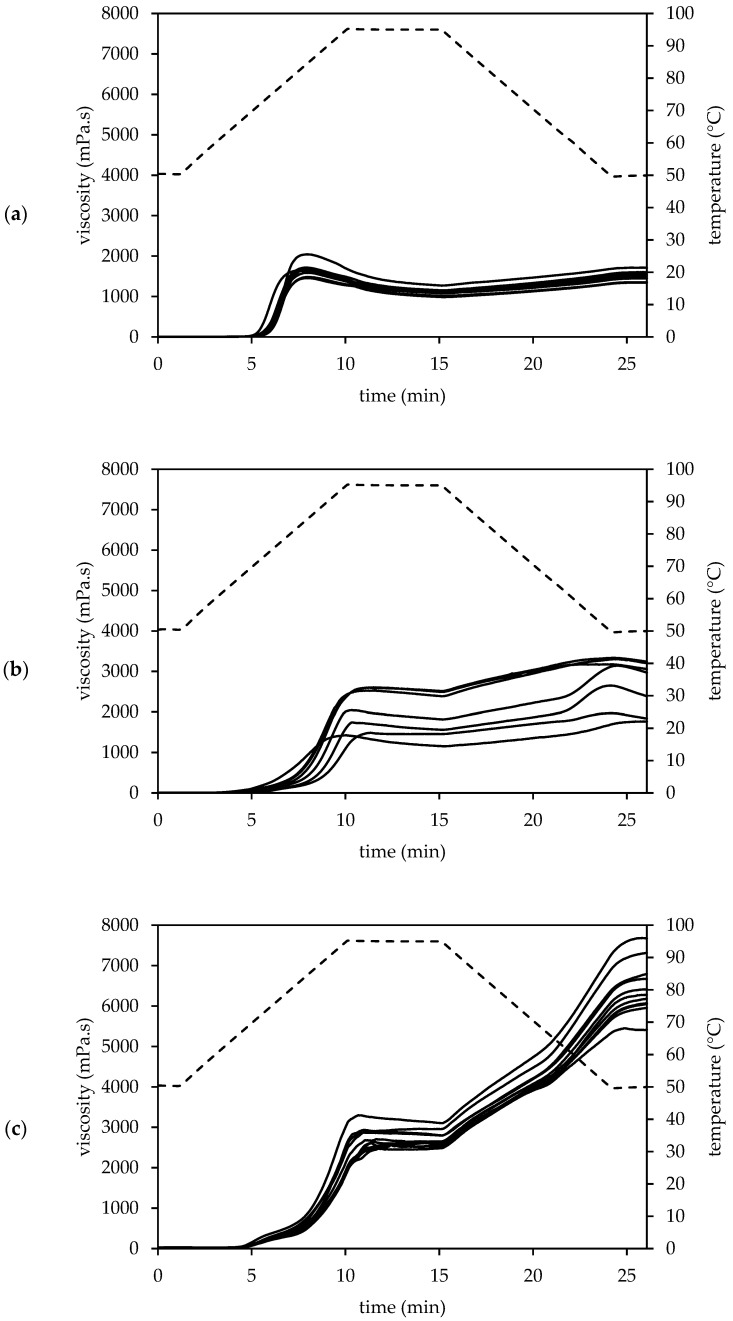
Pasting behaviour of amaranth (**a**, *n* = 8), quinoa (**b**, *n* = 7) and buckwheat (**c**, *n* = 10) wholemeal flour.

The pasting profiles of amaranth, quinoa and buckwheat WMF (Figure 2) were characterised by the pasting parameters in Table S2 (Supplementary Materials). Amaranth WMF had the highest pasting temperature which varied between 65.09 and 68.89 °C. This pasting temperature increased along with the TDF content of the amaranth WMF (*r* = 0.900, *p* = 0.002), which might be attributed to the competition for hydration between dietary fibre and starch [5]. The amaranth WMFs with a high swelling power at 65 °C were characterised by a lower pasting temperature (*r* = −0.895, *p* = 0.006) due to their ability to swell more freely [5]. A similar correlation was found between the swelling power at 55 °C and the pasting temperature of quinoa (*r* = −0.895, *p* = 0.006) and buckwheat WMFs (*r* = −0.723, *p* = 0.016). Quinoa and buckwheat WMF had a lower pasting temperature of 62.22 ± 2.95 and 65.08 ± 0.63 °C, respectively.

Despite the high swelling capacity, amaranth WMFs had the lowest peak viscosity (1657 ± 180 mPa.s). The presence of amylose-lipid complexes is crucial for the heat and shear force resistance of swollen starch granules. The effect of amylose-lipid complexes is limited for amaranth WMFs due to the low amylose content. This leads to high shear sensitivity and low peak viscosity [5]. The higher peak viscosity of AM7 (2045 mPa.s) suggests a higher amylose content as viscosity is directly affected by the amylose content [48]. The low TDF content of AM5 resulted in a lower peak time (7.491 min) and temperature (82.19 °C). Kaur et al. [8] evaluated the pasting parameters of forty-eight *A. hypochondriacus* lines and eleven *A. caudatus* lines and found a peak viscosity between 879 and 1613 mPa.s.

The peak viscosity strongly varied among the quinoa samples and ranged from 1418 to 2606 mPa.s. Other studies also reported a great diversity in the peak viscosity of quinoa. According to Li et al. [21], the peak viscosity of seven quinoa WMFs ranged between 2904 and 5820 mPa.s. Wu et al. [30] studied eleven quinoa varieties and two commercial quinoa samples and found a peak viscosity between 708 and 2364 mPa.s. The diversity in peak viscosity might be attributed to a wide variation in the amylose content [89]. The pasting profile of quinoa WMF showed a less distinct peak, especially if compared to amaranth WMF. Contreras-Jiménez et al. [67] compared the pasting profile of quinoa flour with that of its isolated starch and concluded that the starch developed a significantly different and higher viscosity peak. Authors attributed this to the higher fat content in the flour which produced a more fluid system with lower viscosity. Within the present study, the peak viscosity negatively correlated with the TDF (*r* = −0.920, *p* = 0.003) and K (*r* = −0.756, *r* = 0.049) content of the WMFs, indicating that other non-starch components also affected the pasting properties.

Buckwheat WMF had a significantly higher peak viscosity (2771 ± 242 mPa.s) compared to the other pseudocereals, probably due to the higher starch and amylose content [6]. Buckwheat samples with a higher starch content were generally characterised by a higher peak viscosity (*r* = 0.822, *p* = 0.004), while the protein content had a negative effect on this pasting property (*r* = −0.798, *p* = 0.006). Furthermore, the peak viscosity positively correlated with the Mn (*r* = 0.747, *p* = 0.013) and Zn (*r* = 0.892, *p* = 0.001) content. Bhinder et al. [20] mentioned a flatter peak in the pasting profile of buckwheat and considered this an indication of a low degree of shear-thinning. A similar peak was observed in the present study.

Amaranth WMFs were characterised by the lowest holding strength (1107 ± 87 mPa.s). The pasting profile showed a strong viscosity decrease after reaching maximum, indicating that amaranth WMFs were less resistant to shearing. Quinoa and buckwheat WMFs showed higher shear stability due to the stabilizing effect of amylose-lipid complexes [5]. Holding strength ranged between 1156 and 2513 mPa.s for quinoa, while Wu et al. [30] reported a holding strength between 600 and 2016 mPa.s. Holding strength negatively correlated with the TDF content of the quinoa WMF (*r* = −0.870, *p* = 0.011). Buckwheat showed the highest shear stability as viscosity showed a minor decrease or even slight increase (BU1, BU2, BU3) during the holding phase. Therefore, the holding strength of buckwheat samples was calculated as the average viscosity at the end of the holding phase. The holding strength ranged between 2494 and 3113 mPa.s and correlated with the starch (*r* = 0.788, *p* = 0.007) and protein (*r* = −0.791, *p* = 0.009) content.

The viscosity increased upon cooling as a result of the re-association of the starch granules [8,52]. The final viscosity of amaranth WMF (1502 ± 124 mPa.s) was eventually lower compared to the peak viscosity, as also reported by Kaur et al. [8]. The total setback of amaranth WMFs was low (395 ± 42 mPa.s), indicating a low rate of starch retrogradation [52]. The final viscosity (2677 ± 647 mPa.s) and total setback (767 ± 261 mPa.s) was higher for quinoa WMFs. Wu et al. [30] reported a final viscosity of 672 to 2436 mPa.s and a total setback between −744 and 876 mPa.s for quinoa. Li et al. [21] estimated the total setback between 1068 and 2352 mPa.s. Final viscosity negatively correlated with the TDF content (*r* = −0.961, *p* = 0.001). Dietary fibre is known to retard starch retrogradation. Lipids may also retard retrogradation due to interactions with amylopectin [21].

Cooling of the paste caused a strong viscosity increase for buckwheat as reflected in the high total setback (3702 ± 493 mPa.s). The final viscosity (6421 ± 673 mPa.s) was eventually two to three times higher compared to quinoa. This short-term development of gel structure is attributed to the retrogradation of amylose so the high viscosity increase is expected for buckwheat WMF. The retrogradation of amylopectin occurs at a much slower rate as it involves the re-association of the branch chains [5]. A lower final viscosity (620–2959 mPa.s) and total setback (184–1736 mPa.s) was found by Bhinder et al. [20].

## 4. Conclusions

Different commercial amaranth, quinoa and buckwheat samples were evaluated and their seed characteristics, chemical composition and technological properties were compared. The differences in the seed structure and morphology are reflected in the macronutrient composition of the different pseudocereals. The levels of starch, fat, dietary fibre and minerals were similar in amaranth and quinoa seeds. These seeds have a similar structure in which the embryo is large and surrounds the starchy perisperm. Nevertheless, the protein content is higher in amaranth. The high starch content in buckwheat is related to the larger proportion of the endosperm material. Due to the high amount of starch, buckwheat seeds are characterised by the lowest amounts of fat, dietary fibre and minerals. Its protein content ranges between that of amaranth and quinoa. Buckwheat seeds are larger but also more easily reduced in size as the level of starch damage was low compared to amaranth and quinoa seeds.

The lipid fraction of the pseudocereals mostly contained unsaturated fatty acids. Linoleic acid was the most abundant fatty acid in amaranth and quinoa seeds, while oleic acid had the highest prevalence in buckwheat seeds. Palmitic acid was the main saturated fatty acid in the lipid fraction of pseudocereals of which amaranth contained the highest levels. Furthermore, quinoa seeds contained considerable amounts of α-linolenic acid. The highest content of eicosenoic acid was found in buckwheat.

The three pseudocereals contained high levels of P, K and Mg. The most abundant mineral in amaranth seeds was P but similar levels were found in quinoa and buckwheat. The latter also contained a higher amount of K, while amaranth had the highest Mg level. Important amounts of Ca were also found in amaranth.

The highest soluble and bound phenolic contents were found in buckwheat WMF. The soluble phenolic content was low in amaranth WMF but the total content of PhC was similar to that in quinoa WMF. The most prominent soluble phenolic acid was dihydroxybenzoic acid, while vanillic acid was the most concentrated compound in the bound phenolic fraction of the three pseudocereals. The concentrations of the bound PhC were higher when compared to those of the soluble PhC. The share of the bound PhC was also larger in the total phenolic content.

Amaranth WMF was characterised by the highest swelling power but the pasting parameters showed that its starch fraction had a low shear stability. This is attributable to the low amylose content of amaranth starch. Quinoa and buckwheat WMF were more resistant to shear due to the stabilizing effect of amylose-lipid complexes. Furthermore, buckwheat WMF showed a high rate of retrogradation due to its high amylose content. The strong variation in the pasting profiles of the quinoa samples is probably related to variations in the amylose content. Beside the amylose content, other non-starch components (protein, fat, TDF, minerals) are also likely to have an effect on the technological properties of the pseudocereals WMF.

## Figures and Tables

**Table 1:** Overview of amaranth, quinoa and buckwheat samples.

Type	Code	Origin
amaranth	AM1	India
	AM2	India
	AM3	Peru
	AM4	India
	AM5	India
	AM6	Unknown
	AM7	Unknown
	AM8	India
quinoa	QU1	Peru
	QU2	Unknown
	QU3	Belgium
	QU4	Belgium
	QU5	Belgium
	QU6	Bolivia
	QU7	Bolivia
buckwheat	BU1	Unknown
	BU2	Germany
	BU3	China
	BU4	unknown
	BU5	China
	BU6	unknown
	BU7	Czech Republic
	BU8	unknown
	BU9	unknown
	BU10	China

**Table 2:** Characteristics of amaranth (*n* = 8), quinoa (*n* = 7) and buckwheat (*n* = 10) seeds.

Kernel Characteristic ^1,2^	Amaranth	Quinoa	Buckwheat	*p*-Value ^3^
TKW (g)	0.65–0.79	2.19–4.05	17.59–25.94	
	*0.69* ± *0.05 ^a^*	*3.13* ± *0.80 ^b^*	*23.32* ± *3.48 ^c^*	<*0.001*
test weight (kg/hL)	84.0–86.2	66.8–86.1	76.4–80.2	
	*85.2* ± *0.7 ^b^*	*77.0* ± *5.7 ^a^*	*78.6* ± *1.1 ^a^*	<*0.001*
length (mm)	1.14–1.26	1.77–2.37	3.80–4.56	
	*1.20* ± *0.04 ^a^*	*2.09* ± *0.25 ^b^*	*4.29* ± *0.23 ^c^*	<*0.001*
width (mm)	0.99–1.12	1.63–2.18	3.00–3.44	
	*1.05* ± *0.04 ^a^*	*1.91* ± *0.22 ^b^*	*3.28* ± *0.13 ^c^*	<*0.001*
circularity	0.87–0.88	0.82–0.87	0.73–0.79	
	*0.88* ± *0.01 ^c^*	*0.85* ± *0.02 ^b^*	*0.76* ± *0.02 ^a^*	<*0.001*

^1^ Results presented as minimum–maximum, and mean ± standard deviation. ^2^ TKW = thousand kernel weight; ^3^ Average values marked by the same letter (^a–c^) are not statistically different (*p* > 0.05).

**Table 3:** Chemical composition of amaranth (*n* = 8), quinoa (*n* = 7) and buckwheat (*n* = 10) wholemeal flour.

Property ^1,2^	Amaranth	Quinoa	Buckwheat	*p*-Value ^3^
starch (g/100 g dm)	57.3–65.5	53.6–71.6	67.8–78.3	
	*61.0* ± *2.9 ^a^*	*65.1* ± *6.3 ^a^*	*70.5* ± *3.1 ^b^*	<*0.001*
protein (g/100 g dm)	15.1–16.4	9.5–16.7	13.9–16.4	
	*16.0* ± *0.5 ^b^*	*13.1* ± *2.2 ^a^*	*15.2* ± *0.7 ^a^*	*0.005*
fat (g/100 g dm)	6.47–7.25	2.74–7.34	3.43–3.86	
	*6.81* ± *0.29 ^b^*	*6.36* ± *1.65 ^b^*	*3.68* ± *0.13 ^a^*	<*0.001*
TDF (g/100 g dm)	6.53–11.16	7.15–15.31	3.55–5.86	
	*9.45* ± *2.46 ^b^*	*10.41* ± *3.04 ^b^*	*4.93* ± *0.68 ^a^*	<*0.001*
ash (g/100 g dm)	2.23–2.87	1.92–3.46	1.91–2.30	
	*2.53* ± *0.20 ^b^*	*2.62* ± *0.59 ^b^*	*2.12* ± *0.13 ^a^*	*0.001*

^1^ Results presented as minimum–maximum, and mean ± standard deviation. ^2^ dm = dry matter, TDF = total dietary fibre; ^3^ Average values marked by the same letter (^a,b^) are not statistically different (*p* > 0.05).

**Table 4:** Fatty acid composition (% of total fatty acid methyl esters) of amaranth (*n* = 8), quinoa (*n* = 7) and buckwheat (*n* = 10) wholemeal flour.

Fatty Acid (%) ^1^	Amaranth	Quinoa	Buckwheat	*p*-Value ^2^
C14:0	0.18–0.23	0.15–0.22	0.12–0.15	
	*0.22* ± *0.02 ^b^*	*0.17* ± *0.03 ^b^*	*0.13* ± *0.01 ^a^*	<*0.001*
C14:1	0.12–0.25	0.10–0.48	0.19–0.41	
	*0.18* ± *0.04 ^a^*	*0.18* ± *0.14 ^ab^*	*0.28* ± *0.07 ^b^*	*0.012*
C16:0	18.8–19.4	9.3–10.7	13.4–14.9	
	*18.9* ± *0.2 ^c^*	*9.8* ± *0.5 ^a^*	*14.3* ± *0.6 ^b^*	<*0.001*
C16:1 c	0.09–0.10	0.06–0.11	0.14–0.18	
	*0.10* ± *0.00 ^a^*	*0.08* ± *0.02 ^a^*	*0.16* ± *0.01 ^b^*	<*0.001*
C18:0	3.16–3.67	0.55–0.76	1.71–1.98	
	*3.53* ± *0.17 ^c^*	*0.68* ± *0.08 ^a^*	*1.86* ± *0.08 ^b^*	<*0.001*
C18:1 c9	22.4–25.4	20.2–27.0	32.7–35.2	
	*23.1* ± *1.0 ^a^*	*23.8* ± *3.2 ^a^*	*33.9* ± *0.8 ^b^*	<*0.001*
C18:2 n-6	44.3–47.0	46.4–54.2	34.9–38.8	
	*46.3* ± *1.0 ^b^*	*49.7* ± *3.5 ^b^*	*36.4* ± *1.3 ^a^*	<*0.001*
C18:3 n-3	0.91–1.06	3.71–8.10	2.20–2.56	
	*0.96* ± *0.05 ^a^*	*6.76* ± *1.58 ^c^*	*2.38* ± *0.10 ^b^*	<*0.001*
C20:1	0.26–0.41	1.45–1.72	2.73–2.96	
	*0.30* ± *0.05 ^a^*	*1.57* ± *0.11 ^b^*	*2.85* ± *0.08 ^c^*	*0.001*
C22:1	0.00–0.05	1.21–1.96	0.16–0.21	
	*0.01* ± *0.02 ^a^*	*1.51* ± *0.32 ^c^*	*0.19* ± *0.02 ^b^*	<*0.001*

^1^ Results presented as minimum–maximum, and mean ± standard deviation. ^2^ Average values marked by the same letter (^a–c^) are not statistically different (*p* > 0.05).

**Table 5:** Mineral composition of amaranth (*n* = 8), quinoa (*n* = 7) and buckwheat (*n* = 10) wholemeal flour.

Mineral (mg/kg dm) ^1,2^	Amaranth	Quinoa	Buckwheat	*p*-Value ^3^
Ca	1818–2060	347–1041	151–254	
	*1930* ± *79 ^c^*	*576* ± *288 ^b^*	*220* ± *28 ^a^*	<*0.001*
Cu	1.37–5.46	5.52–11.52	3.41–6.41	
	*4.22* ± *1.21 ^a^*	*7.34* ± *2.32 ^b^*	*4.96* ± *0.88 ^ab^*	*0.002*
Fe	76.8–96.9	33.7–129.9	18.5–38.4	
	*82.6* ± *6.2 ^b^*	*67.9* ± *37.4 ^ab^*	*32.6* ± *5.5 ^a^*	<*0.001*
K	4389–5218	6181–12453	2130–5768	
	*4740* ± *260 ^a^*	*8906* ± *2369 ^b^*	*5174* ± *1079 ^a^*	*0.003*
Mg	2567–2849	1222–2824	1016–2752	
	*2755* ± *100 ^b^*	*2054* ± *529 ^a^*	*2439* ± *510 ^ab^*	*0.017*
Mn	27.0–38.1	16.1–44.9	6.2–21.2	
	*30.0* ± *3.6 ^b^*	*27.6* ± *12.7 ^ab^*	*15.3* ± *4.0 ^a^*	<*0.001*
Na	47.2–91.5	73.1–204.5	36.0–100.4	
	*75.7* ± *14.6 ^ab^*	*127.1* ± *51.2 ^b^*	*65.0* ± *16.9 ^a^*	*0.028*
P	4433–5889	2513–5738	1687–5515	
	*5011* ± *477 ^a^*	*4522* ± *1002 ^a^*	*4640* ± *1083 ^a^*	*0.551*
Zn	31.1–47.7	25.0–44.3	9.4–41.7	
	*35.5* ± *5.5 ^b^*	*36.0* ± *6.2 ^b^*	*26.1* ± *9.1 ^a^*	*0.015*

^1^ Results presented as minimum–maximum, and mean ± standard deviation. ^2^ dm = dry matter; ^3^ Average values marked by the same letter (^a–c^) are not statistically different (*p* > 0.05).

**Table 6:** Phenolic content (mg GAE/g dm) of amaranth (*n* = 8), quinoa (*n* = 7) and buckwheat (*n* = 10) wholemeal flour.

Phenolic Content (mg GAE/g dm) ^1,2^	Amaranth	Quinoa	Buckwheat	*p*-Value ^3^
soluble	0.05–0.07	0.31–0.37	0.48–1.28	
	*0.06* ± *0.01 ^a^*	*0.33* ± *0.02 ^b^*	*1.08* ± *0.23 ^c^*	<*0.001*
bound	1.92–2.91	2.26–3.97	3.06–3.68	
	*2.53* ± *0.34 ^a^*	*2.83* ± *0.61 ^ab^*	*3.36* ± *0.17 ^b^*	<*0.001*
total	1.98–2.98	2.61–4.29	4.15–4.66	
	*2.59* ± *0.34 ^a^*	*3.16* ± *0.61 ^a^*	*4.45* ± *0.19 ^b^*	<*0.001*

^1^ Results presented as minimum–maximum, and mean ± standard deviation. ^2^ GAE = gallic acid equivalents, dm = dry matter; ^3^ Average values marked by the same letter (^a–c^) are not statistically different (*p* > 0.05).

**Table 7:** Contents of soluble phenolic compounds (mg/g dm) in amaranth (*n* = 8), quinoa (*n* = 7) and buckwheat (*n* = 10) wholemeal flour.

Soluble PC (mg/g dm) ^1,2^	Amaranth	Quinoa	Buckwheat	*p*-Value ^3^
gallic acid	0.010–0.017	0.009–0.014	0.007–0.010	
	*0.014* ± *0.003 ^ab^*	*0.012* ± *0.002 ^b^*	*0.008* ± *0.001 ^a^*	*0.001*
dihydroxybenzoic acid	0.135–0.169	0.008–0.109	0.012–0.038	
	*0.151* ± *0.013 ^b^*	*0.056* ± *0.034 ^a^*	*0.028* ± *0.008 ^a^*	< *0.001*
vanillic acid	0.004–0.005	0.008–0.021	0.008–0.009	
	*0.005* ± *0.000 ^a^*	*0.016* ± *0.006 ^a^*	*0.008* ± *0.000 ^a^*	*0.264*
caffeic acid	0.003–0.005	0.003–0.017	n.d.	
	*0.004* ± *0.001 ^a^*	*0.008* ± *0.008 ^a^*	*n.d.*	*0.579*
o-coumaric acid	n.d.	n.d.	0.031–0.091	
	*n.d.*	*n.d.*	*0.071* ± *0.019 ^a^*	*-*
rutin	n.d.	0.033–0.110	n.d.	
	*n.d.*	*0.065* ± *0.025*	*n.d.*	*-*

^1^ Results presented as minimum–maximum, and mean ± standard deviation; ^2^ PC = phenolic compounds, dm = dry matter, n.d. = not detected; ^3^ Average values marked by the same letter (^a,b^) are not statistically different (*p* > 0.05).

**Table 8:** Contents of bound phenolic compounds (mg/g dm) in amaranth (*n* = 8), quinoa (*n* = 7) and buckwheat (*n* = 10) wholemeal flour.

Bound PC (mg/g dm) ^1,2^	Amaranth	Quinoa	Buckwheat	*p*-Value ^3^
vanillic acid	0.247–0.564	0.248–0.621	0.160–0.334	
	*0.447* ± *0.102 ^b^*	*0.342* ± *0.158 ^a^*	*0.228* ± *0.065 ^a^*	<*0.001*
caffeic acid	n.d.	0.111–0.157	0.108–0.119	
	*n.d.*	*0.136* ± *0.019 ^a^*	*0.113* ± *0.007 ^a^*	*0.499*
o-coumaric acid	n.d.	n.d.	0.191–0.223	
	*n.d.*	*n.d.*	*0.205* ± *0.009*	*-*

^1^ Results presented as minimum–maximum, and mean ± standard deviation; ^2^ PC = phenolic compounds, dm = dry matter, n.d. = not detected; ^3^ Average values marked by the same letter (^a,b^) are not statistically different (*p* > 0.05).

**Table 9:** Level of α-amylase and starch damage, and water absorption of amaranth (*n* = 8), quinoa (*n* = 7) and buckwheat (*n* = 10) wholemeal flour.

Property ^1,2^	Amaranth	Quinoa	Buckwheat	*p*-Value ^3^
α-amylase (CU/g dm)	0.06–0.22	0.05–1.07	0.03–0.10	
	*0.15* ± *0.06 ^b^*	*0.25* ± *0.38 ^ab^*	*0.05* ± *0.02 ^a^*	*0.005*
starch damage (% dm)	3.10–3.95	3.51–4.44	0.94–1.43	
	*3.57* ± *0.28 ^b^*	*4.04* ± *0.35 ^c^*	*1.22* ± *0.16 ^a^*	<*0.001*
water absorption (g/g)	1.86–2.15	1.52–2.05	1.59–1.77	
	*2.02* ± *0.09 ^b^*	*1.68* ± *0.18 ^a^*	*1.67* ± *0.06 ^a^*	<*0.001*

^1^ Results presented as minimum–maximum, and mean ± standard deviation. ^2^ dm = dry matter, CU = Ceralpha Unit; ^3^ Average values marked by the same letter (^a–c^) are not statistically different (*p* > 0.05).

## Data Availability

The data presented in this study are available in the article and supplementary material.

## Supplementary Materials

The following are available online at https://www.mdpi.com/2304-8158/10/3/651/s1, Table S1: Swelling power (g/g) of amaranth (*n* = 8), quinoa (*n* = 7) and buckwheat (*n* = 10) wholemeal flour measured at different temperatures (55, 65, 75, 85 and 95 °C). Table S2: Pasting parameters of amaranth (*n* = 8), quinoa (*n* = 7) and buckwheat (*n* = 10) wholemeal flour.

## References

[1] Sedej I., Sakač M., Mandić A., Mišan A., Tumbas V., Hadnađev M., Hadnadev M. (2011). Assessment of antioxidant activity and rheological properties of wheat and buckwheat milling fractions. J. Cereal Sci..

[2] Zanoletti M., Marti A., Marengo M., Iametti S., Pagani M.A., Renzetti S. (2017). Understanding the influence of buckwheat bran on wheat dough baking performance: Mechanistic insights from molecular and material science approaches. Food Res. Int..

[3] Pereira E., Encina-Zelada C., Barros L., Gonzales-Barron U., Cadavez V., Ferreira I.C.F.R. (2019). Chemical and nutritional characterization of Chenopodium quinoa Willd (quinoa) grains: A good alternative to nutritious food. Food Chem..

[4] Bojórquez-Velázquez E., Velarde-Salcedo A.J., De León-Rodríguez A., Jimenez-Islas H., Pérez-Torres J.L., Herrera-Estrella A., Espitia-Rangel E., Barba de la Rosa A.P. (2018). Morphological, proximal composition, and bioactive compounds characterization of wild and cultivated amaranth (*Amaranthus* spp.) species. J. Cereal Sci..

[5] Srichuwong S., Curti D., Austin S., King R., Lamothe L., Gloria-Hernandez H. (2017). Physicochemical properties and starch digestibility of whole grain sorghums, millet, quinoa and amaranth flours, as affected by starch and non-starch constituents. Food Chem..

[6] Haros C.M., Schoenlechner R., Schönlechner R. (2017). Pseudocereals: Chemistry and Technology.

[7] Iglesias-Puig E., Monedero V., Haros M. (2015). Bread with whole quinoa flour and bifidobacterial phytases increases dietary mineral intake and bioavailability. LWT Food Sci. Technol..

[8] Kaur S., Singh N., Rana J.C. (2010). *Amaranthus hypochondriacus* and *Amaranthus caudatus* germplasm: Characteristics of plants, grain and flours. Food Chem..

[9] Aderibigbe O.R., Ezekiel O.O., Owolade S.O., Korese J.K., Sturm B., Hensel O. (2020). Exploring the potentials of underutilized grain amaranth (*Amaranthus* spp.) along the value chain for food and nutrition security: A review. Crit. Rev. Food Sci. Nutr..

[10] Zhu F. (2016). Buckwheat starch: Structures, properties, and applications. Trends Food Sci. Technol..

[11] Alvarez-Jubete L., Arendt E.K.K., Gallagher E. (2010). Nutritive value of pseudocereals and their increasing use as functional gluten-free ingredients. Trends Food Sci. Technol..

[12] Thoufeek Ahamed N., Singhai R.S., Kulkarni P.R., Pal M. (1998). A lesser-known grain, Chenopodium quinoa: Review of the chemical composition of its edible parts. Food Nutr. Bull..

[13] Qin P., Wang Q., Shan F., Hou Z., Ren G. (2010). Nutritional composition and flavonoids content of flour from different buckwheat cultivars. Int. J. Food Sci. Technol..

[14] Hager A.S., Wolter A., Jacob F., Zannini E., Arendt E.K. (2012). Nutritional properties and ultra-structure of commercial gluten free flours from different botanical sources compared to wheat flours. J. Cereal Sci..

[15] De la Barca A.M.C., Rojas-Martínez M.E., Islas-Rubio A.R., Cabrera-Chávez F. (2010). Gluten-Free Breads and Cookies of Raw and Popped Amaranth Flours with Attractive Technological and Nutritional Qualities. Plant Foods Hum. Nutr..

[16] Landi N., Ruocco M.R., Ragucci S., Aliotta F., Nasso R., Pedone P.V., Di Maro A. (2021). Quinoa as source of type 1 ribosome inactivating proteins: A novel knowledge for a revision of its consumption. Food Chem..

[17] Deng Y., Padilla-Zakour O., Zhao Y., Tao S. (2015). Influences of High Hydrostatic Pressure, Microwave Heating, and Boiling on Chemical Compositions, Antinutritional Factors, Fatty Acids, In Vitro Protein Digestibility, and Microstructure of Buckwheat. Food Bioprocess Technol..

[18] Alegbejo J.O. (2013). Nutritional Value and Utilization of Amaranthus (*Amaranthus* Spp.)—A Review. Bayero J. Pure Appl. Sci..

[19] Filho A.M.M., Pirozi M.R., Borges J.T.D.S., Pinheiro Sant’Ana H.M., Chaves J.B.P., Coimbra J.S.D.R. (2017). Quinoa: Nutritional, functional, and antinutritional aspects. Crit. Rev. Food Sci. Nutr..

[20] Bhinder S., Kaur A., Singh B., Yadav M.P., Singh N. (2020). Proximate composition, amino acid profile, pasting and process characteristics of flour from different Tartary buckwheat varieties. Food Res. Int..

[21] Li G., Zhu F. (2017). Physicochemical properties of quinoa flour as affected by starch interactions. Food Chem..

[22] Englyst H.N., Kingman S.M., Cummings J.H. (1992). Classification and measurement of nutritionally important starch fractions. Eur. J. Clin. Nutr..

[23] FAO (1973). Energy and Protein Requirements.

[24] Folch J., Lees M., Sloane Stanley G.H. (1957). A simple method for the isolation and purification of total lipides from animal tissues. J. Biol. Chem..

[25] Raes K., De Smet S., Demeyer D. (2001). Effect of double-muscling in Belgian Blue young bulls on the intramuscular fatty acid composition with emphasis on conjugated linoleic acid and polyunsaturated fatty acids. Anim. Sci..

[26] Shumoy H., Gabaza M., Vandevelde J., Raes K. (2017). Soluble and bound phenolic contents and antioxidant capacity of tef injera as affected by traditional fermentation. J. Food Compos. Anal..

[27] Shumoy H., Raes K. (2016). Antioxidant potentials and phenolic composition of tef varieties: An indigenous ethiopian cereal. Cereal Chem..

[28] Hellemans T., Abera G., De Leyn I., Van der Meeren P., Dewettinck K., Eeckhout M., De Meulenaer B., Van Bockstaele F. (2017). Composition, Granular Structure, and Pasting Properties of Native Starch Extracted from *Plectranthus edulis* (*Oromo dinich*) Tubers. J. Food Sci..

[29] Preetham Kumar K.V., Dharmaraj U., Sakhare S.D., Inamdar A.A. (2016). Preparation of protein and mineral rich fraction from grain amaranth and evaluation of its functional characteristics. J. Cereal Sci..

[30] Wu G., Morris C.F., Murphy K.M. (2014). Evaluation of texture differences among varieties of cooked Quinoa. J. Food Sci..

[31] Konishi Y., Hirano S., Tsuboi H., Wada M. (2004). Distribution of Minerals in Quinoa (Chenopodium quinoa Willd.) Seeds. Biosci. Biotechnol. Biochem..

[32] Prado F.E., Fernández-Turiel J.L., Tsarouchi M., Psaras G.K., González J.A. (2014). Variation of seed mineral concentrations in seven quinoa cultivars grown in two agroecological sites. Cereal Chem..

[33] Wang C.L., Ding M.Q., Zou C.Y., Zhu X.M., Tang Y., Zhou M.L., Shao J.R. (2017). Comparative Analysis of Four Buckwheat Species Based on Morphology and Complete Chloroplast Genome Sequences. Sci. Rep..

[34] Stempinska K., Soral-Smietana M. (2006). Skladniki chemiczne i ocena fizykochemiczna ziarniakow gryki—Porownanie trzech polskich odmian. Żywność Nauka Technol. Jakość..

[35] Temesgen A., Bultosa G. (2017). Physicochemical Characteristics and Nutrient Composition of Three Grain Amaranth Species Grown in Hirna, Eastern Ethiopia. East Afr. J. Sci..

[36] Yamazaki W.T., Briggle L.W. (1969). Components of Test Weight in Soft Wheat 1. Crop Sci..

[37] Abalone R., Cassinera A., Gastón A., Lara M.A. (2004). Some physical properties of amaranth seeds. Biosyst. Eng..

[38] Vilche C., Gely M., Santalla E. (2003). Physical properties of quinoa seeds. Biosyst. Eng..

[39] Quequeto W.D., Siqueira V.C., Schoeninger V., Martins E.A.S., Isquierdo E.P., Da Silva F.P. (2018). Physical properties of buckwheat (*Fagopyrum esculentum* Moench) grains during convective drying. Rev. Bras. Eng. Agric. Ambient..

[40] Gargiulo L., Grimberg Å., Repo-Carrasco-Valencia R., Carlsson A.S., Mele G. (2019). Morpho-densitometric traits for quinoa (*Chenopodium quinoa* Willd.) seed phenotyping by two X-ray micro-CT scanning approaches. J. Cereal Sci..

[41] Parde S.R., Johal A., Jayas D.S., White N.D.G. (2003). Physical properties of buckwheat cultivars. Can. Biosyst. Eng..

[42] Zhu X., Guo W., Wang S. (2013). Sensing moisture content of buckwheat seed from dielectric properties. Trans. ASABE.

[43] Medina W., Skurtys O., Aguilera J.M. (2010). Study on image analysis application for identification Quinoa seeds (*Chenopodium quinoa* Willd) geographical provenance. LWT Food Sci. Technol..

[44] Torbica A., Hadnadev M., Dapčević Hadnadev T. (2012). Rice and buckwheat flour characterisation and its relation to cookie quality. Food Res. Int..

[45] Mota C., Santos M., Mauro R., Samman N., Matos A.S., Torres D., Castanheira I. (2016). Protein content and amino acids profile of pseudocereals. Food Chem..

[46] Unal H., Izli G., Izli N., Asik B.B. (2017). Comparison of some physical and chemical characteristics of buckwheat (*Fagopyrum esculentum* Moench) grains. CYTA J. Food.

[47] Vojtíšková P., Švec P., Kubán V., Krejzová E., Bittová M., Krácmar S., Svobodová B. (2014). Chemical composition of buckwheat plant parts and selected buckwheat products. Potravinarstvo.

[48] Repo-Carrasco-Valencia R., Hellström J.K., Pihlava J.M., Mattila P.H. (2010). Flavonoids and other phenolic compounds in Andean indigenous grains: Quinoa (*Chenopodium quinoa*), kañiwa (*Chenopodium pallidicaule*) and kiwicha (*Amaranthus caudatus*). Food Chem..

[49] Pellegrini M., Lucas-Gonzales R., Ricci A., Fontecha J., Fernández-López J., Pérez-Álvarez J.A., Viuda-Martos M. (2018). Chemical, fatty acid, polyphenolic profile, techno-functional and antioxidant properties of flours obtained from quinoa (*Chenopodium quinoa* Willd) seeds. Ind. Crops Prod..

[50] Dziadek K., Kopeć A., Pastucha E., Piątkowska E., Leszczyńska T., Pisulewska E., Witkowicz R., Francik R. (2016). Basic chemical composition and bioactive compounds content in selected cultivars of buckwheat whole seeds, dehulled seeds and hulls. J. Cereal Sci..

[51] Khan F., Arif M., Khan T.U., Khan M.I., Bangash J.A. (2013). Nutritional Evaluation of Common Buckwheat of Four Different Villages of Gilgit-Baltistan. APRN J. Agric. Biol. Sci..

[52] Menegassi B., Pilosof A.M.R., Arêas J.A.G. (2011). Comparison of properties of native and extruded amaranth (*Amaranthus cruentus* L.—BRS Alegria) flour. LWT Food Sci. Technol..

[53] Tosi E.A., Ré E., Lucero H., Masciarelli R. (2001). Dietary fiber obtained from amaranth (*Amaranthus cruentus*) grain by differential milling. Food Chem..

[54] Capriles V.D.D., Coelho K.D.D., Guerra-Matias A.C.C., Arêas J.A.G.A.G. (2008). Effects of processing methods on amaranth starch digestibility and predicted glycemic index. J. Food Sci..

[55] Ramos-Diaz J.M., Kirjoranta S., Tenitz S., Penttilä P.A., Serimaa R., Lampi A.M., Jouppila K., Ramos Diaz J.M., Kirjoranta S., Tenitz S. (2013). Use of amaranth, quinoa and kañiwa in extruded corn-based snacks. J. Cereal Sci..

[56] Lu L., Murphy K., Baik B.K. (2013). Genotypic variation in nutritional composition of buckwheat groats and husks. Cereal Chem..

[57] Nascimento A.C., Mota C., Coelho I., Gueifão S., Santos M., Matos A.S., Gimenez A., Lobo M., Samman N., Castanheira I. (2014). Characterisation of nutrient profile of quinoa (*Chenopodium quinoa*), amaranth (*Amaranthus caudatus*), and purple corn (*Zea mays* L.) consumed in the North of Argentina: Proximates, minerals and trace elements. Food Chem..

[58] Nasir M.A., Pasha I., Butt M.S., Nawaz H. (2015). Biochemical characterization of quinoa with special reference to its protein quality. Pak. J. Agric. Sci..

[59] Repo-Carrasco-Valencia R.A.-M.M., Serna L.A. (2011). Quinoa (*Chenopodium quinoa*, Willd.) as a source of dietary fiber and other functional components. Cienc. Tecnol. Aliment..

[60] Jahaniaval F., Kakuda Y., Marcone M.F. (2000). Fatty acid and triacylglycerol compositions of seed oils of five *Amaranthus* accessions and their comparison to other oils. J. Am. Oil Chem. Soc..

[61] Tang Y., Li X., Chen P.X., Zhang B., Hernandez M., Zhang H., Marcone M.F., Liu R., Tsao R. (2015). Characterisation of fatty acid, carotenoid, tocopherol/tocotrienol compositions and antioxidant activities in seeds of three *Chenopodium quinoa* Willd. genotypes. Food Chem..

[62] Ryan E., Galvin K., O’Connor T.P., Maguire A.R., O’Brien N.M. (2007). Phytosterol, squalene, tocopherol content and fatty acid profile of selected seeds, grains, and legumes. Plant Foods Hum. Nutr..

[63] Hlinková A., Bednárová A., Havrlentová M., Šupová J., Čičová I. (2013). Evaluation of fatty acid composition among selected amaranth grains grown in two consecutive years. Biologia.

[64] Mota C., Nascimento A.C., Santos M., Delgado I., Coelho I., Rego A., Matos A.S., Torres D., Castanheira I. (2016). The effect of cooking methods on the mineral content of quinoa (*Chenopodium* quinoa), amaranth (*Amaranthus* sp.) and buckwheat (*Fagopyrum esculentum*). J. Food Compos. Anal..

[65] Pongrac P., Vogel-Mikuš K., Jeromel L., Vavpetič P., Pelicon P., Kaulich B., Gianoncelli A., Eichert D., Regvar M., Kreft I. (2013). Spatially resolved distributions of the mineral elements in the grain of tartary buckwheat (*Fagopyrum tataricum*). Food Res. Int..

[66] Egli I., Davidsson L., Juillerat M.A., Barclay D., Hurrell R.F. (2002). The influence of soaking and germination on the phytase activity and phytic acid content of grains and seeds potentially useful for complementary feeding. J. Food Sci..

[67] Contreras-Jiménez B., Torres-Vargas O.L., Rodríguez-García M.E. (2019). Physicochemical characterization of quinoa (*Chenopodium quinoa*) flour and isolated starch. Food Chem..

[68] Miranda-Ramos K.C., Sanz-Ponce N., Haros C.M. (2019). Evaluation of technological and nutritional quality of bread enriched with amaranth flour. LWT Food Sci. Technol..

[69] Beniwal S.K., Devi A., Sindhu R. (2019). Effect of grain processing on nutritional and physico-chemical, functional and pasting properties of amaranth and quinoa flours. Indian J. Tradit. Knowl..

[70] Abderrahim F., Huanatico E., Segura R., Arribas S., Gonzalez M.C., Condezo-Hoyos L. (2015). Physical features, phenolic compounds, betalains and total antioxidant capacity of coloured quinoa seeds (*Chenopodium quinoa* Willd.) from Peruvian Altiplano. Food Chem..

[71] Alvarez-Jubete L., Wijngaard H., Arendt E.K., Gallagher E. (2010). Polyphenol composition and in vitro antioxidant activity of amaranth, quinoa buckwheat and wheat as affected by sprouting and baking. Food Chem..

[72] Guo X.D., Ma Y.J., Parry J., Gao J.M., Yu L.L., Wang M. (2011). Phenolics content and antioxidant activity of tartary buckwheat from different locations. Molecules.

[73] Paśko P., Sajewicz M., Gorinstein S., Zachwieja Z. (2008). Analysis of selected phenolic acids and flavonoids in *Amaranthus cruentus* and *Chenopodium quinoa* seeds and sprouts by HPLC. Acta Chromatogr..

[74] Barba de la Rosa A.P., Fomsgaard I.S., Laursen B., Mortensen A.G., Olvera-Martínez L., Silva-Sánchez C., Mendoza-Herrera A., González-Castañeda J., De León-Rodríguez A. (2009). Amaranth (*Amaranthus hypochondriacus*) as an alternative crop for sustainable food production: Phenolic acids and flavonoids with potential impact on its nutraceutical quality. J. Cereal Sci..

[75] Martín-García B., Pasini F., Verardo V., Gómez-Caravaca A.M., Marconi E., Caboni M.F. (2019). Distribution of free and bound phenolic compounds in buckwheat milling fractions. Foods.

[76] Elgeti D., Nordlohne S.D., Föste M., Besl M., Linden M.H., Heinz V., Jekle M., Becker T. (2014). Volume and texture improvement of gluten-free bread using quinoa white flour. J. Cereal Sci..

[77] Mäkinen O.E., Zannini E., Arendt E.K. (2013). Germination of Oat and Quinoa and Evaluation of the Malts as Gluten Free Baking Ingredients. Plant Foods Hum. Nutr..

[78] Aluwi N.A., Murphy K.M., Ganjyal G.M. (2017). Physicochemical characterization of different varieties of quinoa. Cereal Chem..

[79] Nie Phiarais B.P., Wijngaard H.H., Arendt E.K. (2005). The impact of kilning on enzymatic activity of buckwheat malt. J. Inst. Brew..

[80] Raikos V., Neacsu M., Russell W., Duthie G. (2014). Comparative study of the functional properties of lupin, green pea, fava bean, hemp, and buckwheat flours as affected by pH. Food Sci. Nutr..

[81] Collar C., Angioloni A. (2014). Pseudocereals and teff in complex breadmaking matrices: Impact on lipid dynamics. J. Cereal Sci..

[82] Khan R., Dutta A. (2018). Effect of popping on physico-chemical and nutritional parameters of amaranth grain. J. Pharmacogn. Phytochem..

[83] Tanimola A.R., Otegbayo B., Akinoso R. (2016). Chemical, functional, rheological and sensory properties of amaranth flour and amaranth flour based paste. Afr. J. Food Sci..

[84] Doğan I.S. (2003). Effect of α-amylases on dough properties during Turkish hearth bread production. Int. J. Food Sci. Technol..

[85] Adebowale K.O., Adeniyi Afolabi T., Lawal O.S. (2002). Isolation, chemical modification and physicochemical characterisation of Bambarra groundnut (*Voandzeia subterranean*) starch and flour. Food Chem..

[86] Li J.Y., Yeh A.I. (2001). Relationships between thermal, rheological characteristics and swelling power for various starches. J. Food Eng..

[87] Qian J., Rayas-Duarte P., Grant L. (1998). Partial characterization of buckwheat (*Fagopyrum esculentum*) starch. Cereal Chem..

[88] Siwatch M., Yadav R.B., Yadav B.S. (2019). Chemical, physicochemical, pasting and microstructural properties of amaranth (*Amaranthus hypochondriacus*) flour as affected by different processing treatments. Qual. Assur. Saf. Crops Foods.

[89] Li G., Wang S., Zhu F. (2016). Physicochemical properties of quinoa starch. Carbohydr. Polym..

