# Supplementary material for: Comparison of the Chemical and Technological Characteristics of Wholemeal Flours Obtained from Amaranth (Amaranthus sp.), Quinoa (Chenopodium quinoa) and Buckwheat (Fagopyrum sp.) Seeds
Source: Foods. 2021 Mar 19;10(3):651. doi: 10.3390/foods10030651 (PMC8003493; doi:10.3390/foods10030651)
Supplement: Supplementary file 1 [file foods-10-00651-s001.pdf]

foods

Supplementary Materials

Table S1. Swelling power (g/g) of amaranth (*n* = 8), quinoa (*n* = 7) and buckwheat (*n* = 10) wholemeal flour measured at different temperatures (55, 65, 75, 85 and 95 °C).

Swelling Power (g/g) [1]	Amaranth	Quinoa	Buckwheat	*p*-Value [2]
55 °C	2.66 - 2.97	2.87 - 4.07	2.65 - 3.01	
	2.77 ± 0.10[a]	*3.33 ± 0.47[b]*	*2.84 ± 0.12[ab]*	*0.028*
65 °C	2.56 - 4.33	3.89 - 5.19	3.35 - 5.08	
	3.19 ± 0.56[a]	*4.54 ± 0.53[b]*	*4.39 ± 0.51[b]*	*< 0.001*
75 °C	10.21 – 12.00	5.11 - 6.62	5.37 - 5.81	
	10.87 ± 0.59[b]	*5.86 ± 0.46[a]*	*5.68 ± 0.14[a]*	*< 0.001*
85 °C	11.64 - 14.73	6.11 - 8.47	5.45 - 6.64	
	12.97 ± 1.19[c]	*7.14 ± 0.81[b]*	*6.10 ± 0.38[a]*	*< 0.001*
95 °C	10.77 - 12.11	8.11 - 9.75	6.82 - 8.06	
	11.50 ± 0.42[c]	*8.71 ± 0.50[b]*	*7.30 ± 0.35[a]*	*< 0.001*

[1] Results presented as minimum – maximum, and mean ± standard deviation. [2] Average values marked by the same letter (a-c) are not statistically different ($p > 0.05$).

Table S2. Pasting parameters of amaranth (*n* = 8), quinoa (*n* = 7) and buckwheat (*n* = 10) wholemeal flour.

Pasting Parameter [1]	Amaranth	Quinoa	Buckwheat	*p*-Value [3]
pasting temperature (°C)	65.09 – 68.89	57.64 – 65.46	64.60 – 66.23	
	67.53 ± 1,27[c]	*62.22 ± 2.95[a]*	*65.08 ± 0.63[b]*	*< 0.001*
peak viscosity (mPa.s)	1459 – 2045	1418 - 2606	2539 - 3304	
	1657 ± 180[a]	*2064 ± 524[a]*	*2771 ± 242[b]*	*< 0.001*
peak time (min)	7.491 - 8.127	10.022 - 11.778	10.734 - 12.700	
	7.902 ± 0.182[a]	*10.909 ± 0.692[b]*	*11.473 ± 0.598[b]*	*< 0.001*
peak temperature (°C)	82.19 - 85.36	94.86 - 95.17	95.02 - 95.13	
	84.25 ± 0.91[a]	*95.07 ± 0.11[b]*	*95.08 ± 0.03[b]*	*< 0.001*
holding strength (mPa.s)	987 – 1271	1156 - 2513	2494 - 3113 [2]	
	1107 ± 87[a]	*1910 ± 552[b]*	*2719 ± 201[c]*	*< 0.001*
final viscosity (mPa.s)	1345 – 1714	1756 - 3274	5415 - 7661	
	1502 ± 124[a]	*2677 ± 647[b]*	*6421 ± 673[c]*	*< 0.001*
total setback (mPa.s)	340 – 449	425 - 1224	2806 - 4548	
	395 ± 42[a]	*767 ± 261[b]*	*3702 ± 493[c]*	*< 0.001*

[1] Results presented as minimum – maximum, and mean ± standard deviation. [2] The holding strength of buckwheat samples was calculated as the average viscosity at the end of the holding phase. [3] Average values marked by the same letter (a-c) are not statistically different ($p > 0.05$).
